# Diagnostic and prognostic microRNAs in the serum of breast cancer patients measured by droplet digital PCR

**DOI:** 10.1186/s40364-015-0037-0

**Published:** 2015-06-06

**Authors:** Alessandra Mangolini, Manuela Ferracin, Maria Vittoria Zanzi, Elena Saccenti, Sayda Omer Ebnaof, Valentina Vultaggio Poma, Juana M. Sanz, Angela Passaro, Massimo Pedriali, Antonio Frassoldati, Patrizia Querzoli, Silvia Sabbioni, Paolo Carcoforo, Alan Hollingsworth, Massimo Negrini

**Affiliations:** Department of Morphology, Surgery and Experimental Medicine, University of Ferrara, Via Luigi Borsari, 46, 44121 Ferrara, Italy; Laboratory for Technologies of Advanced Therapies (LTTA), University of Ferrara, Ferrara, Italy; Department of Life Sciences and Biotechnology, University of Ferrara, Ferrara, Italy; Department of Medical Sciences, University of Ferrara, Ferrara, Italy; Oncology Unit, University Hospital of Ferrara, Ferrara, Italy; Mercy Cancer Resource Center/Women’s Center, Oklahoma City, Oklahoma USA

**Keywords:** Breast cancer, Circulating miRNAs, Diagnostic markers, Prognostic markers, Droplet digital PCR

## Abstract

**Background:**

Breast cancer circulating biomarkers include carcinoembryonic antigen and carbohydrate antigen 15–3, which are used for patient follow-up. Since sensitivity and specificity are low, novel and more useful biomarkers are needed. The presence of stable circulating microRNAs (miRNAs) in serum or plasma suggested a promising role for these tiny RNAs as cancer biomarkers. To acquire an absolute concentration of circulating miRNAs and reduce the impact of preanalytical and analytical variables, we used the droplet digital PCR (ddPCR) technique.

**Results:**

We investigated a panel of five miRNAs in the sera of two independent cohorts of breast cancer patients and disease-free controls. The study showed that miR-148b-3p and miR-652-3p levels were significantly lower in the serum of breast cancer patients than that in controls in both cohorts. For these two miRNAs, the stratification of breast cancer patients versus controls was confirmed by receiver operating characteristic curve analyses. In addition, we showed that higher levels of serum miR-10b-5p were associated with clinicobiological markers of poor prognosis.

**Conclusions:**

The study revealed the usefulness of the ddPCR approach for the quantification of circulating miRNAs. The use of the ddPCR quantitative approach revealed very good agreement between two independent cohorts in terms of comparable absolute miRNA concentrations and consistent trends of dysregulation in breast cancer patients versus controls. Overall, this study supports the use of the quantitative ddPCR approach for monitoring the absolute levels of diagnostic and prognostic tumor-specific circulating miRNAs.

**Electronic supplementary material:**

The online version of this article (doi:10.1186/s40364-015-0037-0) contains supplementary material, which is available to authorized users.

## Introduction

Breast cancer is the most frequent cancer and the second leading cause of cancer death among women in industrialized countries. Approximately 1.3 million women develop breast cancer every year [1]. Advances in early diagnosis and treatments have contributed to the decrease of mortality rates over the years. The overall 5-year survival is 90 % when breast cancer is diagnosed at an early stage as opposed to 20 % if disease has spread to distant organs [2]. Physical examination, mammography, and biopsy are the current approaches to breast cancer diagnosis [3]. Carcinoembryonic antigen and carbohydrate antigen 15–3 are circulating tumor markers that are mainly used for patient follow-up [4]. However, the sensitivity of these markers is low, thus calling attention to the need for novel and more accurate noninvasive diagnostic biomarkers.

Studies on circulating microRNAs (miRNAs) opened potential opportunities for the discovery of new tumor biomarkers. miRNAs are a class of small noncoding RNAs that regulate gene expression at the post-transcriptional level [5]. They play a crucial role in the regulation of most, if not all, human genes and their involvement in the deregulation of pathological states such as cancer has been well established [6]. Moreover, miRNAs can be detected in serum or plasma, and their levels may be specifically altered in pathological conditions. Because of their remarkable stability in plasma and serum and the possibility of measuring their levels using noninvasive methods, various studies have suggested a role for circulating miRNAs as novel cancer biomarkers [7–11].

Despite promising results, however, it became evident that several variables (sample collection and storage, RNA purification methods, quantification and normalization methods) could affect final results [12]. In this study, we took advantage of the droplet digital PCR (ddPCR) technique for assessing circulating miRNA levels, an approach that allows absolute quantification without the need for internal/external normalization. Using ddPCR, we investigated five miRNAs in the sera of two independent cohorts of breast cancer patients and disease-free controls to verify whether miRNAs could represent useful diagnostic biomarkers of breast cancer.

## Results

### Circulating miRNAs in sera of breast cancer patients versus healthy controls

We selected five miRNAs (miR-10b-5p, miR-145-5p, miR-148b-3p, miR-425-5p, miR-652-3p) derived from microarray experiments [13] or described in recently published scientific literature as being potential circulating biomarkers (Additional file 1: Table S1). Two independent sets of serum samples from breast cancer patients and disease-free controls were analyzed (Table 1). One group of samples was collected at the University Hospital of Ferrara, Italy, from 2012 to 2014 (cohort A), while the second group of samples was collected at the Mercy’s Woman Center in Oklahoma City, OK, USA, from 2005 to 2013 (cohort B). Serum samples from both cohorts were collected and processed according to the same protocol, and the levels of circulating miRNAs were assessed by ddPCR. This technique allows the measurement of the absolute concentration of circulating miRNAs with no need for a reference gene, a condition particularly important for this type of sample. Hence, miRNA levels were expressed as copies per microliter of serum.

**Table 1:** Clinicopathological features of breast cancer patients

Characteristics		Cohort A (*n* = 28)	Cohort B (*n* = 59)
Age	Mean age (SD)	65.3 (±14.4)	56.7 (±10.4)
	Range	33–91	34–81
Menopausal status	Pre	3 (11 %)	14 (24 %)
	Peri	7 (25 %)	1 (2 %)
	Post	18 (64 %)	44 (75 %)
Histological subtype	Ductal	21 (75 %)	48 (81 %)
	Lobular	4 (14 %)	4 (7 %)
	Tubular	1 (4 %)	1 (2 %)
	Other	2 (7 %)	6 (10 %)
Tumor size (pT)	pT1	21 (75 %)	28 (47 %)
	pT2	7 (25 %)	27 (46 %)
	pT3	0	4 (7 %)
Lymph node involvement (pN)	pN0	21 (75 %)	35 (59 %)
	pN1	6 (21 %)	16 (27 %)
	pN2	0	6 (10 %)
	pN3	1 (4 %)	1 (2 %)
	pNx	0	1 (2 %)
Metastasis (cM)^a^	M0	28 (100 %)	57 (97 %)
	M1	0	2 (3 %)
Stage	I	16 (57 %)	24 (41 %)
	II	11 (39 %)	24 (41 %)
	III	1 (4 %)	9 (15 %)
	IV	0	2 (3 %)
Grade	I	5 (18 %)	11 (19 %)
	II	18 (64 %)	14 (24 %)
	III	5 (18 %)	34 (58 %)
Estrogen receptor	Positive	26 (93 %)	41 (69 %)
	Negative	2 (7 %)	16 (27 %)
	Missing	0	2 (3 %)
Progesterone receptor	Positive	19 (68 %)	35 (59 %)
	Negative	9 (21 %)	22 (37 %)
	Missing	0	2 (3 %)
HER2/*neu* receptor	Positive	3 (11 %)	11 (19 %)
	Negative	25 (89 %)	44 (75 %)
	Uncertain	0	1 (2 %)
	Missing	0	3 (5 %)
Triple negative	ER-/PR-/HER2-	1 (4 %)	9 (15 %)

^a^ *cM* Clinical evidence of metastasis

*HER2/neu* human epidermal growth factor receptor 2, *ER* Estrogen receptor, *PR* Progesterone receptor

**Fig. 1:**
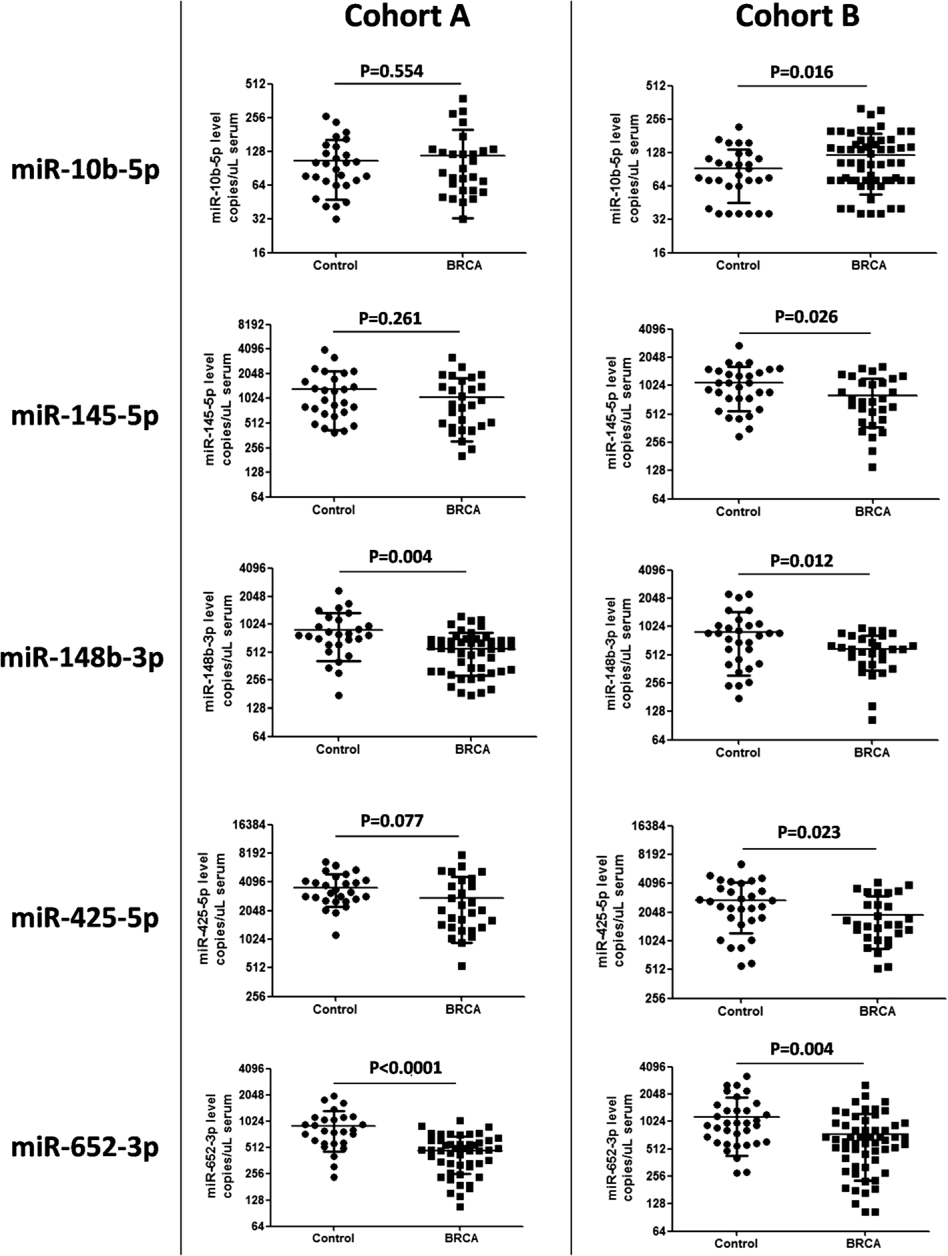
Levels of miRNAs in sera of two independent cohorts of breast cancer and disease-free patients. The level of each miRNA was measured by the ddPCR technique and expressed in copies per microliter in each sample. Each miRNA displays comparable levels and consistent dysregulation in both cohorts. miR-652-3p and miR-148b-3p exhibited a statistically significant reduction in breast cancer patients in both cohorts. The unpaired *t*-test with Welch’s correction was performed to assess significance of differences between patient and control groups. *P*-values of less than 0.05 were deemed to be significant. *BRCA* breast cancer patients

miR-148b-3p and miR-652-3p levels were significantly lower in breast cancer patients than in controls in both cohorts (*p* = 0.0042 and *p* < 0.0001, respectively, in cohort A; *p* = 0.0115 and *p* = 0.0043, respectively, in cohort B) (Fig. 1). miR-145-5p and miR-425-5p were also down-regulated in breast cancer patients compared with controls in both cohorts, but the differences were statistically significant in cohort B only (miR-145-5p: *p* = 0.0257, miR-425-5p: *p* = 0.0226) (Fig. 1). Conversely, miR-10b-5p exhibited a weak increase in cancer patients compared with controls. This trend was statistically significant in cohort B only (*p* = 0.016) (Fig. 1). The reduced representation of high tumor stages was likely responsible for the lack of statistical significance for the results of cohort A (see next section). When combined, the two cohorts produced a highly significant discrimination between cancer patients and controls for all investigated miRNAs, with the strongest discrimination achieved by miR-652 and miR-148b (Additional file 1: Figure S1). To validate these results in comparison with the most commonly used method based on Real Time PCR (RT-PCR) approach, we investigated miR-652 and miR-10b. As RT-PCR normalizer, we employed the non-human Cel-miR-39, which we routinely add at a defined concentration to any serum sample. Results of these analyses confirmed the significant discrimination between samples from breast cancer patients versus controls in the same direction shown by ddPCR-based analyses, thus showing that ddPCR did not introduce any experimental bias if compared to quantitative RT-PCR (Additional file 1: Figure S2).

**Fig. 2:**
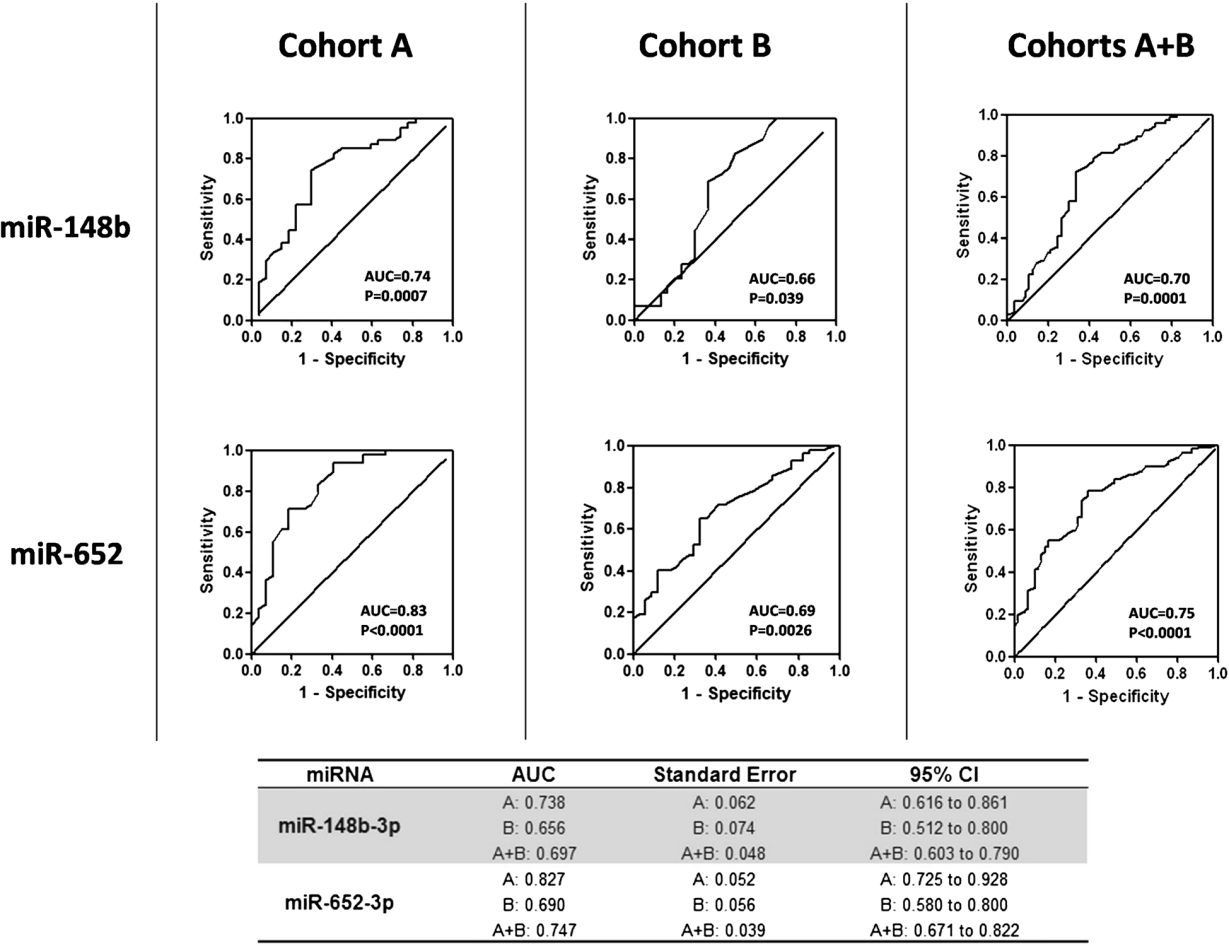
Diagnostic potential of miR-148b and miR-652. Receiver operating characteristic (ROC) curve analyses show that miR-148b and miR-652 exhibit a significant ability to predict breast cancer in both cohorts. Binary logistic regression analysis was performed and ROC curves were generated to evaluate the ability of chosen miRNAs to distinguish cancer patients from controls. *AUC* area under the curve, *CI* confidence interval

Receiver operating characteristic (ROC) curve analysis was performed to evaluate the diagnostic value of the five miRNAs (Fig. 2). miR-652 and miR-148b appeared to represent valuable diagnostic biomarkers. miR-652 was of particular interest because of the highly significant ROC curves in both cohorts. Here, we confirmed a significant lower level of miR-652-3p not only in Luminal A cancer patients (estrogen receptor [ER]/progesterone receptor [PR] positive and human epidermal growth factor receptor 2 [HER2] negative), as previously reported [14]], but also in non-Luminal A cancer patients, versus controls (*p* = 0.020 for Luminal A and *p* = 0.004 for non-Luminal A) (Additional file 1: Figure S3).

### Association of miR-10b with prognostic parameters

**Fig. 3:**
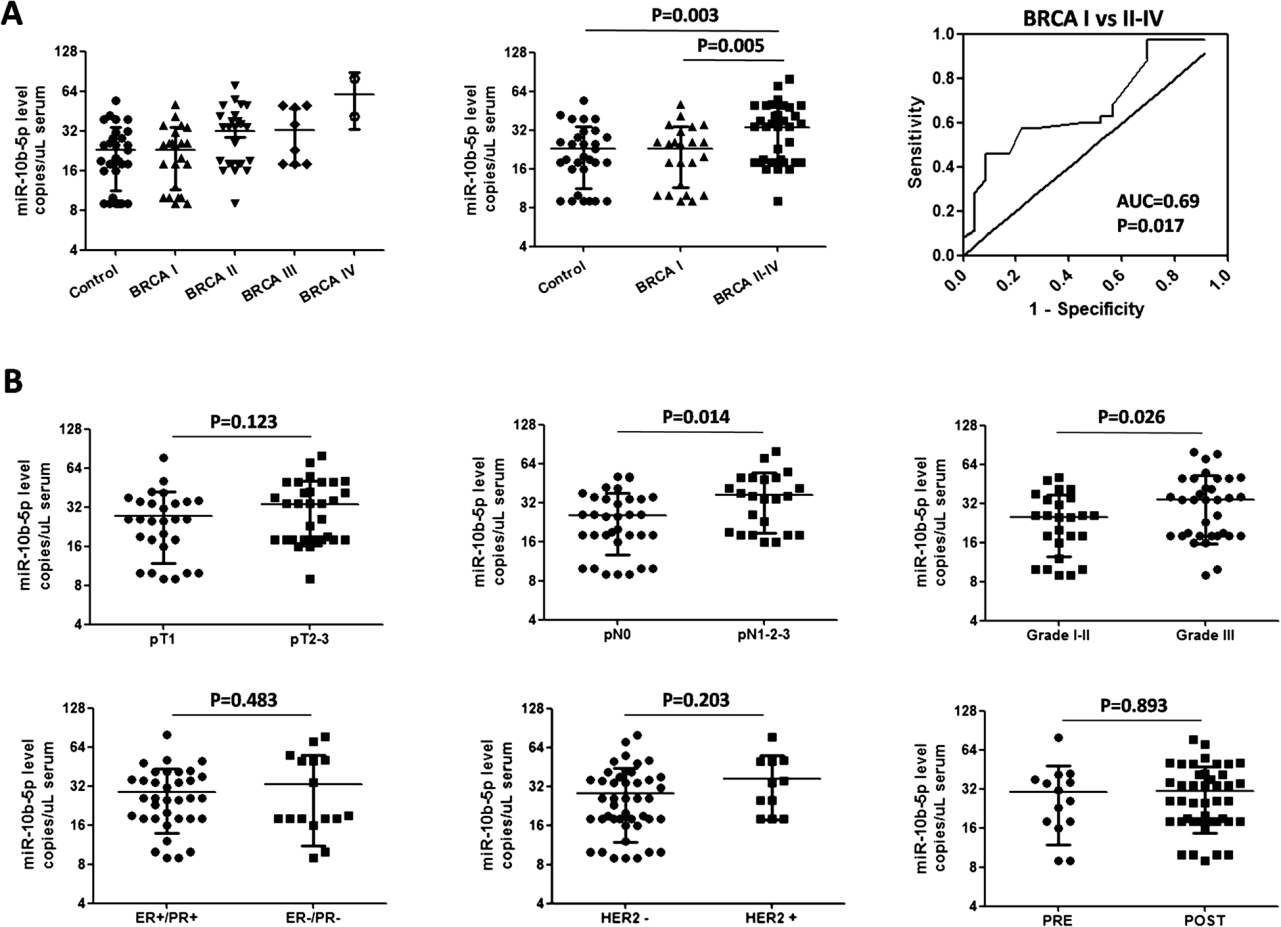
Serum miR-10b-5p increases in patients presenting poor prognostic parameters. **a** Levels of miR-10b increased progressively according to tumor stage. Levels from patients with stages II-IV cancer exhibit a significant difference in comparison to those from patients with stage I cancer (*p* = 0.0047) or controls (*p* = 0.0028). The diagnostic value of miR-10b was assessed by receiver operating characteristic curve analysis. No significant difference was found between stage I and controls. *BRCA* breast cancer patients, *AUC* area under the curve. **b** Higher levels of miR-10b were associated with various clinicopathological parameters: lymph node metastasis (*pN*) (*p* = 0.014) and tumor grade (*p* = 0.026). Albeit not statistically significant, a trend toward increased levels of miR-10b was also detected in cases characterized by increased tumor size (*pT*) or as being human epidermal growth factor receptor 2 positive (HER2+) or estrogen receptor/progesterone receptor negative (ER-/PR-). “PRE” or “POST” refer to the menopausal status

Associations between each miRNA and clinicopathological features were investigated in patients of cohort B (Table 2). Since cohort A included only patients with stage I or II tumors, it was excluded from these analyses. In cohort B, the level of serum miR-10b-5p revealed a concordant increase with tumor stage (Fig. 3). Patients with stage II to IV cancers exhibited significantly higher levels of miR-10b in comparison with patients with a stage I tumor (*p* = 0.0047) or with controls (*p* = 0.0028). Conversely, no significant difference was found between stage I patients and controls.

**Table 2:** miR-10b-5p: copies per microliter of serum according to clinicopathological features

Feature		Cohort A (Italy)	Cohort B (USA)
		Average copies/μL ± SD	Average copies/μL ± SD
		(*n* = number of patients)	(*n* = number of patients)
Tumor size (pT)	pT1	108.7 ± 78.0 (*n* = 19)	98.6 ± 47.3 (*n* = 28)
	pT2	97.3 ± 55.9 (*n* = 6)	143.4 ± 70.3 (*n* = 27)
	pT3	/	75.0 ± 11.9 (*n* = 4)
Lymph node involvement (pN)	pN0	88.7 ± 37.2 (*n* = 18)	101.2 ± 54.6 (*n* = 35)
	pN1-2-3	113.3 ± 89.4 (*n* = 7)	145.2 ± 70.8 (*n* = 23)
Stage	I	82.9 ± 28.5 (*n* = 14)	91.3 ± 45.9 (*n* = 23)
	II	107.7 ± 79.9 (*n* = 10)	128.0 ± 61.8 (*n* = 25)
	III	134.0 (*n* = 1)	130.5 ± 60.2 (*n* = 8)
	IV	/	242.2 ± 110.3 (*n* = 2)
Grade	I	80.8 ± 36.5 (*n* = 5)	114.9 ± 43.2 (*n* = 11)
	II	112.7 ± 88.6 (*n* = 18)	91.2 ± 58.8 (*n* = 13)
	III	105.0 ± 54.3 (*n* = 5)	136.0 ± 74.81 (*n* = 34)
ER/PR status	ER+/PR+	142.1 ± 98.44 (*n* = 17)	115.0 ± 60.1 (*n* = 35)
	ER-/PR-	74.0 (*n* = 1)	131.9 ± 89.4 (*n* = 16)
HER2/*neu* receptor	Positive	103.7 ± 80.8 (*n* = 3)	112.9 ± 65.5 (*n* = 11)
	Negative	115.8 ± 88.4 (*n* = 25)	145.8 ± 75.0 (*n* = 44)
Triple Negatives	ER-/PR-/HER2-	74.0 (*n* = 1)	117.8 ± 90.2 (*n* = 9)
	Others	118.2 ± 85.61 (*n* = 27)	121.5 ± 63.36 (*n* = 49)

*ER* estrogen receptor, *PR* progesterone receptor, *HER2/neu* human epidermal growth factor receptor 2

Notably, miR-10b-5p was also significantly up-regulated in association with other clinicopathological features of prognostic significance, including higher tumor grading and lymph node metastases (Fig. 3). Albeit not statistically significant, the average level of miR-10b-5p was also higher in patients carrying HER2-positive or ER/PR-negative cancers. The number of triple negative breast cancer were too few to lead to any meaningful result.

These data indicate that miR-10b-5p is significantly associated with parameters associated with a poor prognosis. No significant association was found between miR-145-5p, miR-148b-3p, miR-425-5p, or miR-652-3p and any clinicopathological feature.

## Discussion

The presence of stable miRNAs circulating in plasma or serum suggested their potential use as noninvasive biomarkers in cancer patients. In the last few years, several authors have demonstrated that a number of circulating miRNAs could discriminate breast cancer patients from healthy individuals [15, 16] or could be linked to breast cancer subtypes [14, 17]. Unfortunately, the combined effects of several variables made results poorly reproducible and their translation into clinically useful applications not feasible [12, 18–20]. For example, the heterogeneity of investigated populations (age, tumor features), or the differences in sample type (plasma or serum) or sample processing protocols could have been responsible for the apparent discrepancies among the various studies. Moreover, a variety of methods were used to normalize data, thereby producing non-comparable or difficult-to-compare results [12].

Here, we used the ddPCR technique to measure circulating miRNAs. ddPCR is a technique that can achieve absolute quantification of nucleic acids by combining limiting dilutions, end-point PCR, and Poisson statistics. In fact, the partitioning of the PCR reaction into up to 20,000 separate droplets mimics a binary distribution of the target. More important, being an end-point PCR, ddPCR can tolerate wide variations in amplification efficiencies without affecting copy number estimation of the target [21–24].

Using ddPCR, we analyzed the levels of five miRNAs in the serum of two independent cohorts of breast cancer patients and disease-free controls. Blood samples were collected at two independent institutions and processed separately, with no differences in procedures for obtaining serum samples. We analyzed serum, instead of plasma, as it is the most commonly available patient material, and the procedure used to collect serum is homogeneous at different institutions, thus helping to reduce uncertainties in preanalytical procedures. Notably, all of the analyzed miRNAs showed comparable absolute levels in the sera of the two cohorts (see Fig. 1). Most important, both cohorts exhibited consistent trends of dysregulation in breast cancer patients versus controls. The differences between breast cancer cases and controls in cohort B were statistically significant for all five miRNAs, whereas only miR-148b-3p and miR-652-3p reached statistical significance in cohort A, possibly because of differences in clinicopathological characteristics. We performed ROC curve analyses in which we evaluated the possible diagnostic potential of the five circulating miRNAs. Areas under the curves and *P*-values were significant for miR-148b-3p and miR-652-3p in both cohorts (see Fig. 2), suggesting the potential value of these two miRNAs as breast cancer biomarkers.

Our data on circulating miRNAs show both similarities to and discrepancies from those of previous reports (listed in Additional file 1: Table S1). However, differences in experimental settings and technical approaches make it difficult to compare the findings from various reports. Similar to our findings, three previous reports found a decreased level of miR-145-5p in breast cancer patients [17, 25, 26]. The up-regulation of miR-10b-5p in breast cancer patients in our study is also in agreement with several published reports [16, 25, 27]. We did not find significant differences in cohort A. However, since this group consisted of patients carrying stage I or II cancers, this finding does not contradict a positive correlation between miR-10b and more advanced disease; it is also consistent with the results described in a report by Roth et al., who found a higher level of miR-10b in patients with metastatic disease [10]. For mir-652-3p, our results are in agreement with one report that indicated a decreased level in patients with Luminal A-like breast cancer in comparison with controls [14]. Conversely, Cuk et al. showed increased levels of miR-652-3p, likely because the study was performed on plasma instead of serum samples [15]. In support of this suggestion, analysis of a small number (*n* = 20) of plasma samples from our breast cancer cohort revealed that miR-652-3p was indeed increased in breast cancer patients compared with that in controls (data not shown). Finding an opposite trend in serum or plasma samples is not new [12, 13] and raises the question about the different genesis of circulating miRNAs [28]. Concerning miR-148b-3p and miR-425-5p, the published literature reports opposite results to ours, as both miRNAs were found to be higher in breast cancer patients than in healthy controls. However, studies on miR-148b were all performed by using plasma [15, 29, 30], whereas the miR-425 study was performed with serum, but normalization was based on the mean of assays performed on all samples [17]; thus, these results are not directly comparable with ours.

In the search for possible correlations with clinicopathological features, we found that miR-10b-5p levels were increased in the serum of patients with a high cancer stage or grade, or with the presence of lymph node metastases. Albeit not statistically significant, the average expression of miR-10b-5p was also higher in cases with ER/PR-negative tumors and in cases with HER2-positive tumors. Together, these findings indicate that miR-10b-5p represents a biomarker of tumor aggressiveness. This suggestion is also supported by other studies that indicated higher levels of circulating miR-10b in patients with metastatic breast cancer and worsening clinical stage [10, 27, 31]. It is notable that the role of miR-10b in invasion and metastasis has been thoroughly investigated and its importance proven [32–35]. miR-10b has also been found to be highly expressed in the vasculature of high-grade breast cancer [36]. Taken together, these findings suggest that tumor and microenvironment features may be directly responsible for the increased levels of circulating miR-10b in the bloodstream of patients with advanced breast cancer. The possibility of assessing the absolute levels of miR-10b in the serum of patients by using a robust technique such as ddPCR could represent a potential new approach for monitoring disease behavior in breast cancer patients.

## Conclusions

Overall, this study supports the use of the quantitative ddPCR approach for monitoring the absolute levels of specific miRNAs as diagnostic and prognostic serum biomarkers in breast cancer patients.

## Methods

### Study cohorts

Two cohorts of patients were investigated. Serum samples from cohort A (*n* = 55) were collected at the General Surgery Unit of the University Hospital of Ferrara, Italy, from 28 breast cancer patients and 27 age-matched disease-free controls. Serum samples from cohort B (*n* = 94) were collected at the Mercy Women’s Center in Oklahoma City, OK, USA, from 59 breast cancer patients and 35 age-matched controls (Additional file 1: Table S2). Ethical approval was granted by the ethics committees of the respective institutions. Written informed consent was obtained from all individuals enrolled in the study. Table 1 summarizes the clinicopathological features of the patients.

### Sample preparation and RNA purification from serum

Blood samples from cohort A and B were collected in red stopper clot tubes (Greiner Bio-One VACUETTE in cohort A, BD Vacutainer in cohort B) and processed within 1 h; they were centrifuged at 1000 *g* for 10 min at room temperature, and serum was stored at −80 °C in 200 μL aliquots until use. 3 μL of 4.16 nM solution of synthetic miRNA cel-miR-39-3p (ucaccggguguaaaucagcuug) from C.Elegans (synthesized by IDT) was added to each aliquot. Total RNA was isolated from 200 μL of serum by using the MiRNeasy kit (Qiagen). RNA was eluted from spin columns in 35 μL of nuclease-free water.

### Reverse transcription, ddPCR and Real-Time PCR

cDNA was synthesized in a 20 μL reaction by using the Universal cDNA synthesis kit II (Exiqon), starting from 3 μL of RNA according to the manufacturer’s guidelines for serum and plasma samples. Synthesized cDNA was diluted 50-fold, and 8 μL was assayed in a 20 μL PCR reaction volume according to the manufacturer’s protocol for miRCURY LNA Universal RT microRNA PCR (Exiqon) with EvaGreen (Bio-Rad). Each PCR reaction was mixed with 70 μL of droplet generator oil for EvaGreen in a disposable cartridge and applied to the QX200 droplet generator device (Bio-Rad) that portioned each sample into 20,000 nanoliter-sized droplets. Each sample was then transferred into a 96-well PCR plate and PCR was performed to the end point according to the manufacturer’s protocol (Bio-Rad). The same procedure was applied to all test samples and negative controls. At the end of the PCR reaction, the QX200 droplet reader (Bio-Rad) was used to count PCR-positive and PCR-negative droplets: the “singulator” unpacks the emulsified droplets and streams them in a single line past a two-color optical detection system. Positive droplets, which contain at least one copy of the target miRNA, exhibit increased fluorescence compared with negative droplets. The fraction of PCR-positive droplets enables the target to be quantified according to Poisson distribution. Quantitative Real-Time PCR was performed on miR-10b-5p and miR-652-3p using the ExiLent CyberGreen mastermix (Exiqon). Cel-miR-39 was used to normalize miRNAs levels with the 2^-ΔΔCt method.

### Statistical analysis

Statistical analysis was performed by using Prism software version 5.0 (GraphPad, La Jolla, CA). An unpaired *t*-test with Welch’s correction was performed to assess the significance of differences between data distribution. A *p*-value of less than 0.05 was deemed to be significant. Binary logistic regression analysis was performed and ROC curves were generated to evaluate the ability of the chosen miRNAs to discriminate cancer cases versus controls.

## Additional file

Additional file 1: Figure S1. Distribution of miRNA levels in sera of the two combined cohorts of breast cancer and disease-free patients. **Figure S2**. Validation of ddPCR results using Real-Time PCR. **Figure S3**. Serum miR-652-3p is significantly reduced in patients affected by either Luminal A or non-Luminal A breast cancers. **Table S1**. Published data on circulating microRNAs in human breast cancer patients. **Table S2**. Demographic characteristics of study populations.

## Abbreviations

ddPCR: Droplet digital PCR
miRNA: MicroRNA
ER: Estrogen receptor
PR: Progesterone receptor
HER2: Human epidermal growth factor receptor 2
ROC: Receiver operating characteristic
AUC: Area under the curve

## References

[1] Jemal A, Bray F, Center MM, Ferlay J, Ward E, Forman D (2011). Global cancer statistics. CA Cancer J Clin.

[2] Howlader N, Noone A, Krapcho M, Garshell J, Miller D, Altekruse S (2014). SEER Cancer Statistics Review, 1975–2011.
http://seer.cancer.gov/csr/1975_2011/.

[3] Checka CM, Chun JE, Schnabel FR, Lee J, Toth H (2012). The relationship of mammographic density and age: implications for breast cancer screening. AJR Am J Roentgenol.

[4] Harris L, Fritsche H, Mennel R, Norton L, Ravdin P, Taube S (2007). American Society of Clinical Oncology 2007 update of recommendations for the use of tumor markers in breast cancer. J Clin Oncol.

[5] Bartel DP (2004). MicroRNAs: genomics, biogenesis, mechanism, and function. Cell.

[6] Negrini M, Ferracin M, Sabbioni S, Croce CM (2007). MicroRNAs in human cancer: from research to therapy. J Cell Sci.

[7] Mitchell PS, Parkin RK, Kroh EM, Fritz BR, Wyman SK, Pogosova-Agadjanyan EL (2008). Circulating microRNAs as stable blood-based markers for cancer detection. Proc Natl Acad Sci U S A.

[8] Turchinovich A, Weiz L, Langheinz A, Burwinkel B (2011). Characterization of extracellular circulating microRNA. Nucleic Acids Res.

[9] Lawrie CH, Gal S, Dunlop HM, Pushkaran B, Liggins AP, Pulford K (2008). Detection of elevated levels of tumour-associated microRNAs in serum of patients with diffuse large B-cell lymphoma. Br J Haematol.

[10] Roth C, Rack B, Muller V, Janni W, Pantel K, Schwarzenbach H (2010). Circulating microRNAs as blood-based markers for patients with primary and metastatic breast cancer. Breast Cancer Res.

[11] Zhao H, Shen J, Medico L, Wang D, Ambrosone CB, Liu S (2010). A pilot study of circulating miRNAs as potential biomarkers of early stage breast cancer. PLoS One.

[12] Jarry J, Schadendorf D, Greenwood C, Spatz A, van Kempen LC (2014). The validity of circulating microRNAs in oncology: five years of challenges and contradictions. Mol Oncol.

[13] Ferracin M, Lupini L, Salamon I, Saccenti E, Musa G, Zagatti B, et al. Absolute quantification of cell-free microRNAs in cancer patients. Oncotarget 2015, in press. 10.18632/oncotarget.3859 PMC4546486 26036630

[14] McDermott AM, Miller N, Wall D, Martyn LM, Ball G, Sweeney KJ (2014). Identification and validation of oncologic miRNA biomarkers for luminal A-like breast cancer. PLoS One.

[15] Cuk K, Zucknick M, Heil J, Madhavan D, Schott S, Turchinovich A (2013). Circulating microRNAs in plasma as early detection markers for breast cancer. Int J Cancer.

[16] Mar-Aguilar F, Mendoza-Ramirez JA, Malagon-Santiago I, Espino-Silva PK, Santuario-Facio SK, Ruiz-Flores P (2013). Serum circulating microRNA profiling for identification of potential breast cancer biomarkers. Dis Markers.

[17] Kodahl AR, Lyng MB, Binder H, Cold S, Gravgaard K, Knoop AS (2014). Novel circulating microRNA signature as a potential non-invasive multi-marker test in ER-positive early-stage breast cancer: a case control study. Mol Oncol.

[18] Cheng HH, Yi HS, Kim Y, Kroh EM, Chien JW, Eaton KD (2013). Plasma processing conditions substantially influence circulating microRNA biomarker levels. PLoS One.

[19] Mestdagh P, Hartmann N, Baeriswyl L, Andreasen D, Bernard N, Chen C (2014). Evaluation of quantitative miRNA expression platforms in the microRNA quality control (miRQC) study. Nat Methods.

[20] Sourvinou IS, Markou A, Lianidou ES (2013). Quantification of circulating miRNAs in plasma: effect of preanalytical and analytical parameters on their isolation and stability. J Mol Diagn.

[21] Dingle TC, Sedlak RH, Cook L, Jerome KR (2013). Tolerance of droplet-digital PCR vs real-time quantitative PCR to inhibitory substances. Clin Chem.

[22] Hindson CM, Chevillet JR, Briggs HA, Gallichotte EN, Ruf IK, Hindson BJ (2013). Absolute quantification by droplet digital PCR versus analog real-time PCR. Nat Methods.

[23] Miotto E, Saccenti E, Lupini L, Callegari E, Negrini M, Ferracin M (2014). Quantification of circulating mirnas by droplet digital PCR: comparison of evagreen- and taqman-based chemistries. Cancer Epidemiol Biomarkers Prev.

[24] Pinheiro LB, Coleman VA, Hindson CM, Herrmann J, Hindson BJ, Bhat S (2012). Evaluation of a droplet digital polymerase chain reaction format for DNA copy number quantification. Anal Chem.

[25] Chan M, Liaw CS, Ji SM, Tan HH, Wong CY, Thike AA (2013). Identification of circulating microRNA signatures for breast cancer detection. Clin Cancer Res.

[26] Ng EK, Li R, Shin VY, Jin HC, Leung CP, Ma ES (2013). Circulating microRNAs as specific biomarkers for breast cancer detection. PLoS One.

[27] Zhao FL, Hu GD, Wang XF, Zhang XH, Zhang YK, Yu ZS (2012). Serum overexpression of microRNA-10b in patients with bone metastatic primary breast cancer. J Int Med Res.

[28] Etheridge A, Gomes CP, Pereira RW, Galas D, Wang K (2013). The complexity, function and applications of RNA in circulation. Front Genet.

[29] Cuk K, Zucknick M, Madhavan D, Schott S, Golatta M, Heil J (2013). Plasma microRNA panel for minimally invasive detection of breast cancer. PLoS One.

[30] Shen J, Hu Q, Schrauder M, Yan L, Wang D, Medico L (2014). Circulating miR-148b and miR-133a as biomarkers for breast cancer detection. Oncotarget.

[31] Chen W, Cai F, Zhang B, Barekati Z, Zhong XY (2013). The level of circulating miRNA-10b and miRNA-373 in detecting lymph node metastasis of breast cancer: potential biomarkers. Tumour Biol.

[32] Ma L, Reinhardt F, Pan E, Soutschek J, Bhat B, Marcusson EG (2010). Therapeutic silencing of miR-10b inhibits metastasis in a mouse mammary tumor model. Nat Biotechnol.

[33] Ma L, Teruya-Feldstein J, Weinberg RA (2007). Tumour invasion and metastasis initiated by microRNA-10b in breast cancer. Nature.

[34] Biagioni F, Bossel Ben-Moshe N, Fontemaggi G, Yarden Y, Domany E, Blandino G (2013). The locus of microRNA-10b: a critical target for breast cancer insurgence and dissemination. Cell Cycle.

[35] Liu Y, Zhao J, Zhang PY, Zhang Y, Sun SY, Yu SY (2012). MicroRNA-10b targets E-cadherin and modulates breast cancer metastasis. Med Sci Monit.

[36] Plummer PN, Freeman R, Taft RJ, Vider J, Sax M, Umer BA (2013). MicroRNAs regulate tumor angiogenesis modulated by endothelial progenitor cells. Cancer Res.

