# Supplementary material for: Diagnostic and prognostic microRNAs in the serum of breast cancer patients measured by droplet digital PCR
Source: Biomark Res. 2015 Jun 6;3:12. doi: 10.1186/s40364-015-0037-0 (PMC4483205; doi:10.1186/s40364-015-0037-0)
Supplement: Additional file 1: Figure S1. — Distribution of miRNA levels in sera of the two combined cohorts of breast cancer and disease-free patients. Figure S2. Validation of ddPCR results using Real-Time PCR. Figure S3. Serum miR-652-3p is significantly reduced in patients affected by either Luminal A or non-Luminal A breast cancers. Table S1. Published data on circulating microRNAs in human breast cancer patients. Table S2. Demographic characteristics of study populations. [file 40364_2015_37_MOESM1_ESM.pdf]

Additional files

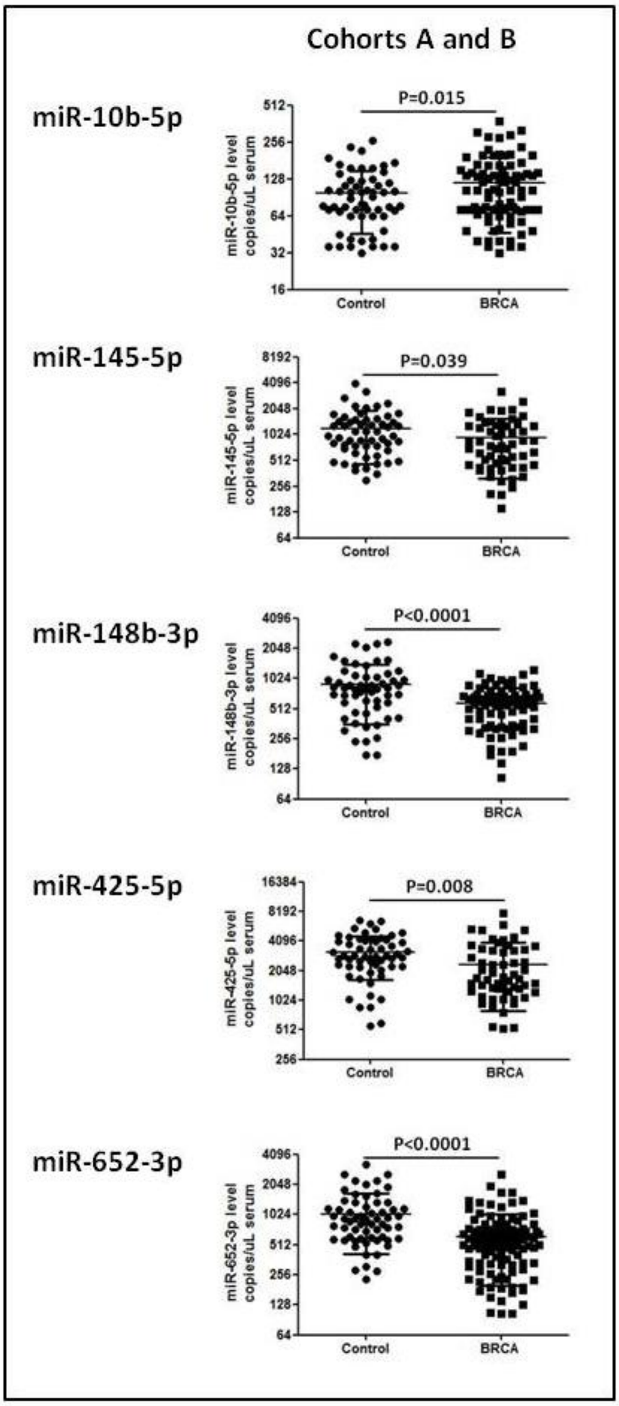

Figure S1. Distribution of miRNA levels in sera of the two combined cohorts of breast cancer and disease-free patients. Results of the two cohorts shown in Figure 1 were combined. The unpaired t-test with Welch's correction was performed to assess significance of differences between breast cancer patients and control groups. P-values of less than 0.05 were deemed to be significant. Significant discrimination between breast cancer patients and disease-free controls as well as trends of dysregulation were all confirmed. BRCA = breast cancer patients

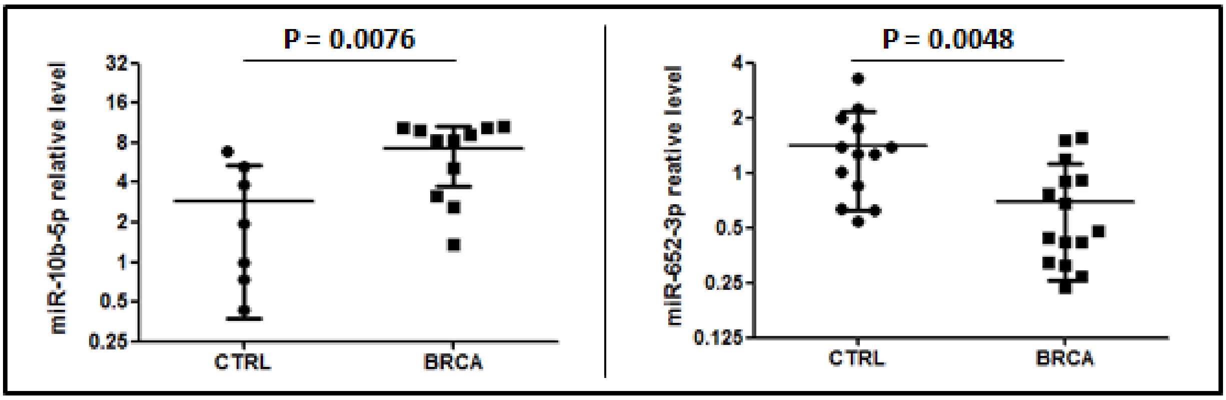

Figure S2. Validation of ddPCR results using Real-Time PCR. The serum levels of miR_10b-5p (left) and miR-652-3p (right) were investigated in a subset of samples from cohort B using Real-Time PCR instead of ddPCR method. The same trend of dysregulation found with ddPCR was also confirmed with this method. Cel-miR-39 was used as standard reference to normalize results as described in manuscript text.

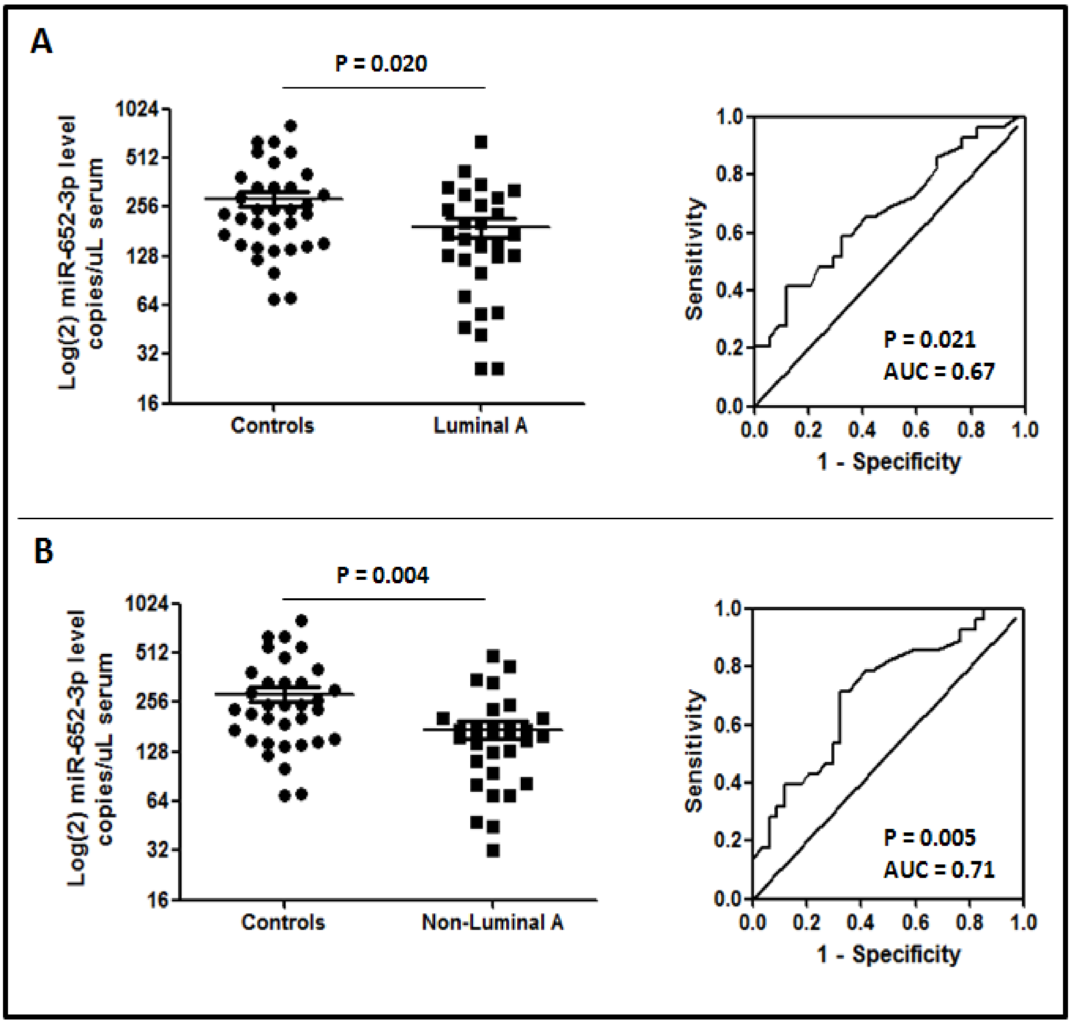

Figure S3. Serum miR-652-3p is significantly reduced in patients affected by either Luminal A or non-Luminal A breast cancers. Analysis of cohort B indicated that the levels of serum miR-652-3p are significantly reduced in Luminal A (ER+/PR+/HER2-) (panel A) and other non-Luminal A cases (panel B) in comparison with disease-free controls. The same results were obtained by analysis of cohort A. AUC = area under the curve.

Table S1. Published data on circulating microRNAs in human breast cancer patients

miRNA	Sample type	Experimental design	Results	References
**miR-10b-5p**	Serum	SyberGreen qRT-PCR<br>Normalizer: miR-16	Higher in BRCA vs healthy controls	(1)
	Serum	TaqMan qRT-PCR<br>Normalizer: 18S RNA	Higher in BRCA vs healthy controls	(2)
	Serum	TaqMan qRT-PCR<br>Normalizer: miR-16	Higher in pM1 BRCA vs pM0 BRCA	(3)
	Serum/plasma	TaqMan qRT-PCR<br>Normalizer: miR-16	No significant difference between BRCA and healthy controls	(4)
	Serum	LNA RT-PCR human miRNA panel (Exiqon)<br>Normalizer: miR-103, miR-191	Higher in BRCA vs healthy controls	(5)
	Serum	LNA-based qRT-PCR<br>QX200 digital PCR system	Higher in BRCA vs healthy controls<br>Higher in BRCA stage-IV vs I<br>Higher in pN + BRCA vs pN0 BRCA<br>Higher in grade III BRCA vs grade I-II BRCA	This paper
**miR-145-5p**	Serum	TaqMan qRT-PCR<br>Normalizer: 18S RNA	Higher in BRCA vs healthy controls	(2)
	Plasma	SyberGreen qRT-PCR<br>Normalizer: RNU6B	Lower in BRCA vs healthy controls	(6)
	Serum	LNA-based qRT-PCR<br>Normalizer: based on the mean of the assays detected in all samples	Lower in BRCA vs healthy controls	(7)
	Serum	LNA-based qRT-PCR<br>QX200 digital PCR system	Lower in BRCA vs healthy controls	This paper
**miR-148b-3p**	Plasma	TaqMan qRT-PCR<br>Normalizer: Spike-in cel-miR-39	Higher in early BRCA vs healthy controls	(8)
	Plasma	TaqMan qRT-PCR<br>Normalizer: Spike-in cel-miR-39	Higher in BRCA vs healthy controls<br>Higher in benign BRCA vs healthy controls	(9)
	Plasma	miRCURY LNA Universal RT microRNA PCR/TaqMan qRT-PCR<br>Normalizer: miR-93	Higher in early BRCA vs healthy controls	(10)
	Serum	LNA-based qRT-PCR<br>QX200 digital PCR system	Lower in BRCA vs healthy controls	This paper
**miR-425-5p**	Serum	LNA-based qRT-PCR	Higher in ER+ BRCA vs healthy controls	(7)

		Normalizer: based on the mean of the assays detected in all samples		
	Serum	LNA-based qRT-PCR<br>QX200 digital PCR system	Lower in BRCA vs healthy controls	This paper
**miR-652-3p**	Blood	TaqMan qRT-PCR<br>Normalizer: miR-16	Lower in Luminal A-like BRCA vs healthy controls	(11)
	Plasma	TaqMan qRT-PCR<br>Normalizer: Spike-in cel-miR-39	Higher in BRCA vs healthy controls<br>Higher in benign BRCA vs healthy controls	(9)
	Serum	LNA-based qRT-PCR<br>QX200 digital PCR system	Lower in BRCA vs healthy controls<br>Lower in Luminal A-like BRCA vs healthy controls	This paper

1. Zhao FL, Hu GD, Wang XF, Zhang XH, Zhang YK, Yu ZS. Serum overexpression of microRNA-10b in patients with bone metastatic primary breast cancer. J Int Med Res 2012; 40:859-66.
2. Mar-Aguilar F, Mendoza-Ramirez JA, Malagon-Santiago I, et al. Serum circulating microRNA profiling for identification of potential breast cancer biomarkers. Dis Markers 2013; 34:163-9.
3. Roth C, Rack B, Muller V, Janni W, Pantel K, Schwarzenbach H. Circulating microRNAs as blood-based markers for patients with primary and metastatic breast cancer. Breast Cancer Res 2010; 12:R90.
4. Heneghan HM, Miller N, Lowery AJ, Sweeney KJ, Newell J, Kerin MJ. Circulating microRNAs as novel minimally invasive biomarkers for breast cancer. Ann Surg 2010; 251:499-505.
5. Chan M, Liaw CS, Ji SM, et al. Identification of circulating microRNA signatures for breast cancer detection. Clin Cancer Res 2013; 19:4477-87.
6. Ng EK, Li R, Shin VY, et al. Circulating microRNAs as specific biomarkers for breast cancer detection. PLoS One 2013; 8:e53141.
7. Kodahl AR, Lyng MB, Binder H, et al. Novel circulating microRNA signature as a potential non-invasive multi-marker test in ER-positive early-stage breast cancer: a case control study. Mol Oncol 2014; 8:874-83.
8. Cuk K, Zucknick M, Heil J, et al. Circulating microRNAs in plasma as early detection markers for breast cancer. Int J Cancer 2013; 132:1602-12.
9. Cuk K, Zucknick M, Madhavan D, et al. Plasma microRNA panel for minimally invasive detection of breast cancer. PLoS One 2013; 8:e76729.
10. Shen J, Hu Q, Schrauder M, et al. Circulating miR-148b and miR-133a as biomarkers for breast cancer detection. Oncotarget 2014; 5:5284-94.
11. McDermott AM, Miller N, Wall D, et al. Identification and validation of oncologic miRNA biomarkers for luminal A-like breast cancer. PLoS One 2014; 9:e87032.

Table S2. Demographic characteristics of study populations

	Cohort A (Italy)		Cohort B (USA)	
	**Cancer patients**	**Controls**	**Cancer patients**	**Controls**
**Total**	28	27	59	35
**Mean age, years (SD)**	65.3 (±14.4)	54.2 (±14.8)	56.73 (±10.4)	53.2 (±11.5)
**Range**	33-91	28-78	34-81	27-94
**< 50**	4 (14.3%)	8 (29.6%)	15 (25.4%)	12 (34.3%)
**50-60**	8 (28.6%)	8 (29.6%)	22 (37.3%)	14 (40.0%)
**60-70**	6 (21.4%)	3 (11.1%)	15 (25.4%)	8 (22.9%)
**> 70**	10 (35.7%)	5 (18.5%)	7 (11.9%)	1 (2.9%)
**not known**	0	3 (11%)	0	0
